# Spatial asymmetry of the paternity success in nests of a fish with alternative reproductive tactics

**DOI:** 10.1038/s41598-021-82508-6

**Published:** 2021-02-04

**Authors:** F. Poli, I. A. M. Marino, M. Santon, E. Bozzetta, G. Pellizzato, L. Zane, M. B. Rasotto

**Affiliations:** 1 Department of Biology, University of Padova, Via U. Bassi 58/B, 35131 Padua, Italy; 2 Consorzio Interuniversitario per le Scienze del Mare (CoNISMa), Piazzale Flaminio 9, 00196 Rome, Italy; 3 Institute for Evolution and Ecology, Department of Biology, University of Tübingen, Auf der Morgenstelle 28, 72076 Tübingen, Germany

**Keywords:** Ecology, Evolution

## Abstract

Guard-sneaker tactics are widespread among fish, where territorial males defend a nest and provide parental care while sneakers try to steal fertilizations. Territorials and sneakers adopt diverse pre- and post-mating strategies, adjusting their ejaculate investment and/or behavioural responses to the presence of competitors. The relative distance of competitors from the spawning female plays a major role in influencing male mating strategies and the resulting paternity share. However, territorial male quality and sneaking intensity do not fully account for the variability in the relative siring success occurring among species. An often neglected factor potentially affecting sneakers proximity to females is the nest structure. We conducted a field experiment using the black goby, whose nests show two openings of different size. We found that territorial males defend more and sneaking pressure is higher at the front, larger access of the nest than at the back, smaller one. Moreover, microsatellite paternity analysis shows that territorials sire more offspring at the back of their nest. Such a predictable spatial distribution of the paternity share suggests that nest structure might work as an indirect cue of male relative siring success, potentially influencing the territorial male investment in parental care and/or the female egg deposition strategy.

## Introduction

Intrasexual competition may lead to the evolution of alternative reproductive tactics (ARTs) in either sex, with male and/or female phenotypes varying in behaviour, morphology, physiology and life history traits within the same population^1,2^. Male ARTs are particularly widespread among teleost fish, where dominant males monopolise most females or resources, while subordinate males sneak in dominant males matings and steal fertilizations^1,3^. Dominant or territorial males feature well developed secondary sexual traits and invest primarily in nest/territory defence, courtship, and often parental care^3^. By contrast, opportunistic/sneaker males always mate in the presence of at least another male and invest predominantly in ejaculate production to engage in sperm competition^4,5^. Not only do they produce more sperm, which can be also faster or more viable in comparison to territorial males, but in some species also produce a seminal fluid that can impair the performance of the sperm of territorial males^3,6,7^. In the most common guard-sneaker type of ARTs^3^, both dominant and opportunistic males flexibly tailor their pre- and/or post- mating effort in relation to the number of competing rivals^8^. For example, sneaker males were shown to strategically modulate the number of sperm released in each mating event according to the number of competitors in two goby species^9^, and territorial males change their ejaculation rate according to the numbers of rivals in the European bitterling, *Rhodeus sericeus*^10^. Territorial males may also adjust their behaviour in relation to the presence of sneakers, both before and after fertilization. Before mating, they generally perform more nest-guarding as competition increases^10,11^. After mating, depending on the certainty of paternity, territorial males can increase parental care efforts towards their own offspring^12–14^ and/or the cannibalism over rivals’ embryos^15–19^.

The pattern emerging from genetic data indicates that pre- and/or post-fertilization male strategies result in an uneven paternity share, skewed towards territorial males and influenced by the number of competitors involved^19–25^. However, the number of sneakers as well as the quality of territorial males do not always fully explain the paternity pattern recorded, suggesting that other factors affect the siring success^19–25^. A crucial role is played by the relative proximity of rival males to the female during spawnings^3^, with the paternity success of territorial males decreasing the closer sneakers can get to the spawning female^24,27–31^. An often-neglected factor influencing sneaker proximity is the structure of the nest/substrate where females lay the eggs. Nest structure is, indeed, known to affect the opportunity for sneaking attempts^32–35^, influencing both the nest-guarding effort and the ejaculate investment of territorial males^31–37^. Moreover, the structure of a nest may result in a different vulnerability of its areas to sneaking attempts. Indeed, differences in the spatial structure of the mating environment may influence territorial male’s defence effort, paternity success, and potentially his post-mating behaviour in terms of parental care and embryos cannibalism^16,34^. However, whether and how nest shape may affect paternity success between sneakers and territorial males has never been reported before. To our knowledge, the only study investigating the spatial distribution of paternity success has been conducted in a few nests of the lingcod, *Ophiodon elongatus*, and it was found that territorial males sired more offspring in the deep and less accessible part of the nest^27^. Lingcod males nest in rocky crevices^27^ whose conformation might allow dominant males to physically exclude other males only from the area further away from the crevice’s entrance, thus suffering a spatially dependent paternity loss.

In our study, we tested the possibility that the paternity success of territorial males varies depending on the characteristics of the nest in the black goby, *Gobius niger* L., a fish with guard-sneaker mating tactics^38,39^. Black goby nests consist of small cavities, dug in the mud along sloping seabed, under rocks or artificial substrates, that usually present a principal front entrance and a secondary smaller access at the back^38,39^. Considering that while performing parental care territorial males fan the eggs to increase the oxygen supply, the small back opening presumably enhances the otherwise poor water circulation inside the nest, favouring its ventilation^38^. In this species the ejaculates of different male phenotypes achieve the same fertilization success when simultaneously released at the same distance from unfertilized eggs^37^. Thus, paternity success of males critically depends on their distance from the spawning female, which might be influenced by the structure of the nest. In particular, we expected that (1) sneaking pressure and territorial nest defence are greater in the front larger access of the nest than at the back smaller one and that (2) paternity success of territorial males is spatially asymmetric, higher in the back of the nest compared to the front. To test our predictions, we quantified (1) the behaviour of territorial and sneaker males, and (2) the distribution of the paternity success along the nest using molecular analysis on embryos sampled from artificial nests placed in the field.

## Materials and methods

**Figure 1:**
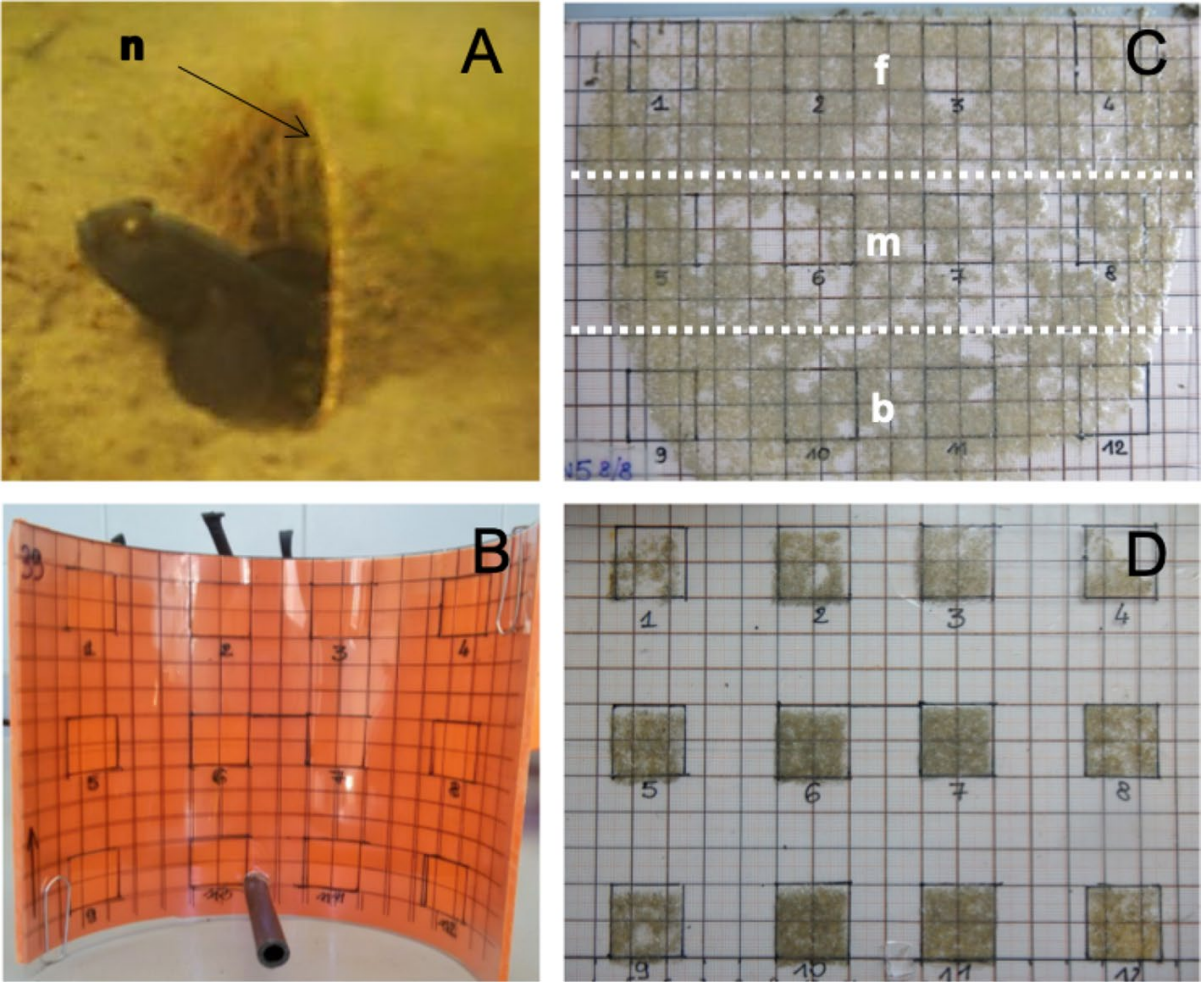
Black goby artificial nest and egg sampling. (**A**) Territorial male inside an artificial nest (n); (**B**) acetate sheet with printed grid fixed on the nest ceiling; (**C**) acetate sheet, with embryos, divided in three equal sized sub-areas from the main to the secondary nest access: (f) front, (m) middle and (b) back; (**D**) acetate sheet sub-areas (1 to 12) were isolated to randomly collect eight embryos from each one.

This study was conducted in the southern part of the Venetian lagoon (North Adriatic Sea; Italy) during the black goby breeding season (May–July 2015–2016)^38,39^. First, we quantified the intensity of the defensive behaviour of territorial males and the sneaking pressure at the front and back nest entrances using behavioural observations on natural nests in the field (45°15′41.80′′ N 12°17′14.27′′ E). Second, we placed artificial nests in this site and in another one (45°14′17.06′′ N 12°15′57.40′′ E) and performed molecular parentage analysis to measure the distribution of the paternity success of territorial males across three areas of equal size within the nest. The area close to the main entrance was named “front area”, the following one “middle area”, while the area facing the back nest opening, in proximity of the muddy slope, was defined as “back area” (Fig. 1).

### Defensive behaviour of territorial males and sneaking pressure

#### Experimental design

Field observations on 44 natural nests were performed by recording with action cameras (GoPro HERO4) the nests and their surroundings (10 cm radius, corresponding to the area where most of the attack-defence events take place). The recordings, one per nest, lasted for 30 min, with the first ten minutes being excluded from the analysis to avoid possible interference caused by the researcher. We measured (1) the time spent by territorial males guarding the front and the back entrances (i.e. overlooking the nest access and chasing the sneakers that move closer), (2) the time spent by sneakers attacking the front and the back entrance of the nests (i.e. moving closer to the nest access enough to try to intrude), (3) mating occurrence and (4) the total number of sneakers and females that were visible around each nest. Females are easily distinguishable from sneakers, despite showing a similar colouration, because of the swollen abdomen: indeed only females ready to spawn approach the nests. On the other hand, territorial males defending nests display the black breeding colouration.

### Distribution of the territorial males’ paternity success

#### Experimental design

We assessed the paternity success of territorial males in the front, middle and back areas in 20 different nests. Artificial nests consisted of a PVC tube cut in half along its long axis in two halves (14 cm × 14 cm × 3.5 cm) (Fig. 1A; see supplementary data SI). Ten artificial nests were placed in each of the two natural breeding sites at 4 m depth and 15 cm distance from each other^40^ with one of the two openings fully accessible (length: 14 cm; height: 3.5 cm), and the other one completely obstructed by insertion into the muddy slope. Nevertheless, all territorial males dug a smaller secondary entrance (diameter: 5.28 cm ± 0.93) on the non-accessible side, creating a structure similar to natural nests^38^. An acetate sheet with a printed grid subdivided in three equal areas (each of 93.33 cm^2^) was placed at the ceiling of each artificial nest. In each area four squared sub-areas of the same size (2 cm^2^) were distributed in a systematic stratified design to allow for homogeneous sampling (Fig. 1B–D). Two days after territorial males had started to guard the nest and to court females and had mated they were caught. They were then anaesthetized in a water solution of MS 222 (Tricaine sulfate, Sandoz), sized (average total length = 11.57 cm ± 1.15; range 10–15 cm) and marked by removing a small fin-clip. The entire process was carried out underwater, and each territorial male was placed back in its nest and observed until completely recovered from anaesthesia. We removed the acetate sheets six days later, when the embryos had reached an adequate developmental stage (eye-stage) to perform DNA analysis. Eight embryos were then randomly sampled in each of the 12 squared sub-areas to do molecular parentage analysis (Fig. 1D). At this stage we took another fin-clip of the territorial males in each nest to control for potential nest-takeover events (i.e. when a territorial takes control of the nest of a rival). Sampling and experimental procedures were approved by the animal ethics committee of the University of Padova (CEASA, permission no. 35/2011) and in compliance with the ARRIVE guidelines (http://www.nc3rs.org.uk/page.asp?id=1357).

#### Microsatellite and parentage analysis

A total of 112 additional adults (territorial N = 60; sneakers N = 19; females N = 33) were obtained from fishers in the same area and sampled to estimate the allele frequency of the population and the power of the parentage analysis of the microsatellites chosen (see below). Individuals were sexed on the basis of the urogenital papilla and male phenotypes categorized according to body size and the dorsal fin elongation^38,39^. They were then anaesthetized (as above), and a small fin-clip was removed to perform the microsatellites molecular analysis. After sampling, all specimens were released unharmed in the field.

Genomic DNA was extracted from adult fin-clips following a salting-out protocol^41^ whilst embryonic DNA was extracted using the Gloor and Engels fast protocol^42^. All individuals were genotyped at four microsatellite loci (Table 1). Forward primers of all four loci were labelled with fluorescent dyes for detection with ABI PRISM automatic sequencer and amplified together in a single 10 μl multiplex PCR reaction using Qiagen Multiplex PCR kit and following manufacturer’s instructions. PCR conditions were as follows: initial activation step for 15 min at 95 °C, 30 cycles of 30 s at 94 °C, 90 s at 57 °C and 1 min at 72 °C, and final elongation for 30 min at 60 °C. PCR products were run at the BMR Genomics Molecular Biology service (www.bmr-genomics.it) on an ABI 3100 automated sequencer and sized with the standard LIZ500 (Applied Biosystems).

**Table 1:** Genetic characterisation of the 4 microsatellite loci used in this study.

Locus name	Repeat motifs	Accession number	5′–3′ primer sequences	Size range (bp)
Zo + + 44m13	(AC)_13_	AY687332	F:GCCGATCGATAGCTCTGACT R:CCTAGATTTCCTCCCCTGGT	192–216
Gn agat 31	(AG)_4_(TG)_9_	JQ731624	F:GCGCGGGTACTAAAATGAGA R:GCGATCCTAAAATGGAGCAC	147–169
Gn ac 24	(TG)_10_	JQ731620	F:TGATAGCACGCAGGAGAGAA R:AGGTTGAGACCGCCATGTAA	105–113
Gn aat 9	(CA)_10_	JQ731631	F:AAACAAACGGGGTCCTATGG R:TTCATTCGTGCCGTTTTACA	111–153

The table shows locus name, repeat motifs, Genbank accession numbers, sequences of forward (F) and reverse (R) primers, allelic size range^44,45^.

Fragment analysis was performed using PEAK SCANNER ver. 1.0 (Applied Biosystems). Ten per cent of all specimens was re-genotyped and rescored manually to detect potential scoring errors. No discrepancies were found. To minimize the negative consequences of a poor allele calling, we automated binning with the software FLEXIBIN ver. 2^43^ and manually checked the final score to ensure the accuracy of the process.

Power of markers for parentage assignment was estimated using two different approaches. The expected exclusion probability, for each locus and for all loci combined, was calculated with the software GERUD 2.0^46^ according to the equations reported in Dodds et al.^47^; the allele frequencies calculated with Genepop version 4.2^48^ from the reference sample of 132 adults collected were used. In addition, probability of identity was calculated, for each locus and for all loci, with the software GENALEX ver. 6^49^ (Table 1).

For the molecular parentage analysis, the embryonic genotypes were manually assigned to the candidate father, which is the territorial male owner of the nest. The paternity success was calculated as the ratio between the number of embryos fertilized by the territorial male over the total number of embryos analysed per nest’s area. We also estimated the minimum number of possible fathers, supposed to be sneaker males, for unassigned embryos, and minimum number of mothers from the embryos assigned to the territorial male, on the basis of the highest alleles number recorded per locus.

### Statistical analysis

We analysed the data using two Generalized Linear Mixed Effects Models with the lme4^50^ and the glmmTMB^51^ packages for R v4.0.3^52^. For the behavioural observations we used a negative binomial distribution (link = log) with time spent by males defending the nest as response variable. Since the time spent by sneakers attacking the nest was correlated with the time spent by the parental males defending it (see Results), we did not fit a second model to analyse sneakers’ attack time. We included as fixed effects the main predictor *nest area* (front or back), the *number of sneakers and of females around the nest* (continuous covariates), and *mating occurrence* (occurring or not during observation time). We instead analysed the fertilization success distribution of territorial males using a binomial distribution (link = logit). The response variable was a two-column integer matrix with number of eggs fertilized by parental males as column 1, and by sneaker males as column 2. The fixed factors of the full model included the main predictor *nest area* (front, middle, back), the *number of sneakers and females around the nest* (continuous covariates), and the *breeding site,* as we compared genetic data from artificial nests distributed in two natural breeding sites (1 and 2 see Material and Methods). We computed VIFs (variance inflation factors) to assess collinearity among covariates, which did not show any issue^53^. Continuous covariates have been standardised to facilitate model conversion. Both models used *Individual nests IDs* as random component to account for the repeated measurements of each nest^54^.

We report a proxy for the goodness-of-fit of each model (conditional R^2^, i.e. the proportion of variation explained by fixed and random components)^55^. We used Wald z-tests to assess the overall significance of fixed effects. To further explore the significance of fixed multi-level factorial predictors, we computed pairwise differences adjusted for multiplicity (Tukey’s HSD) among the different levels of the predictor of interest using the emmeans package for R^56^. Models’ assumptions were verified by checking for overdispersion, fit, number of simulated zeros and by plotting residuals versus fitted values, and versus each covariate present in the models^53^. Means are shown ± standard deviation unless specified otherwise.

### Ethical approval

All animal handling described in the present study was conducted according to European Union guidelines for handling of animals for research purposes (http://ec.europa.eu/environment/chemicals/lab_animals/home_en.htm) and the national guidelines provided by the Institutional OPBA ("Body for the protection of Animal") and Italian Ministry of Health, as per Legislative Decree no. 116/1992 and no. 26/2014. In particular, sampling and experimental procedures were approved by the animal ethics committee of the University of Padova (CEASA, permission no. 35/2011).

## Results

### Defensive behaviour of territorial males and sneaking pressure

**Figure 2:**
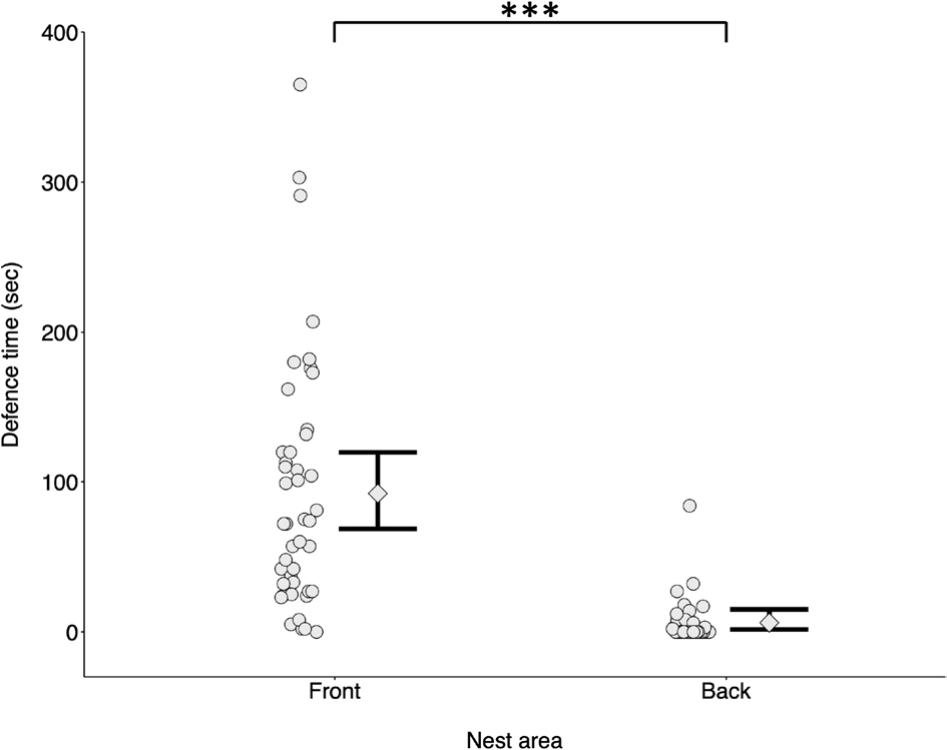
Time spent by territorial males defending the nest as a function of nest area (‘front’, ‘back’). Territorial males spend more time defending the frontal area of the nest. Data points: average male’s defence time for each nest (N = 44). Error bars: model-predicted group medians (diamonds) with 95% credible intervals (vertical bold black lines) computed for each nest area over 10,000 simulations of the model and averaged across number of sneakers, number of females and mating occurrence.

The time that territorial males spent defending the nest was significantly higher in the front area (93.9 ± 83.6 s) compared to the back area (5.32 ± 14.2 s) (GLMM: front-back p < 0.001; N = 44) (Fig. 2, Table 2). Territorial male defending time was significantly correlated with the time spent by sneakers attacking the two nest entrances (Pearson correlation: r = 0.27, p = 0.01). Indeed, sneakers’ attacks also predominantly occurred in the front area of nests (9.43 ± 11.5 s) compared to the back (1.93 ± 5.13 s).

**Table 2:** Statistical analysis: defensive behaviour of territorial males.

Predictors	Predicted mean	Lower 95% CI	Upper 95% CI	P
**Time spent by territorial males defending the nest as a function of nest area** **N = 44 nests, R**^**2**^_**cond**_** = 0.66**				
Intercept (front, no mating)	4.384	4.057	4.384	< 0.001
Nest area (back, no mating)	− 2.640	− 1.940	− 3.340	< 0.001
Number of sneakers (standardised)	0.021	− 0.241	0.283	0.875
Number of females (standardised)	− 0.039	− 0.296	0.218	0.768
Mating occurrence (yes)	0.119	− 0.112	0.902	0.127

Generalized Linear Mixed Model with time spent by territorial males defending the nest as response variable. Predicted means and their credible intervals (CI) are based on a negative binomial distribution with log-link (see Materials and methods). For factorial predictors, estimates are computed using the indicated intercept level as reference. This choice is arbitrary and does not affect overall conclusions.

### Distribution of the paternity success of territorial males

#### Microsatellite and parentage analysis

Molecular analysis showed that the 4 microsatellite loci used were highly polymorphic: number of alleles ranged from 14 to 29 among loci, and observed heterozygosity ranged from 0.576 to 0.900 (Table 3). The combined expected exclusion probabilities for all loci were extremely high, 0.99 (neither parent known), whereas the combined probability of identity for the four loci was significant, indicating that the microsatellite loci in our study had enough power (Table 3). Hardy–Weinberg equilibrium was tested with the software Genepop version 4.2^48^ and the expected and observed heterozygosity with the software Arlequin version 3.5.2.2^58^.

**Table 3:** Summary of genetic diversity at four microsatellite loci selected for parentage analysis in *Gobius niger*.

Locus name	H _*obs*_	H _*exp*_	NA	HWE	Probability of identity	Total exclusion probability neither parent known
Zo + + 44m13	0.823	0.923	29	0.0044	0.012	0.726
Gn agat 31	0.576	0.589	14	0.2871	0.185	0.218
Gn ac 24	0.900	0.943	24	0.0138	0.007	0.782
Gn aat 9	0.780	0.850	15	0.0283	0.041	0.533
Tot	–	–	–	–	6.325 E−07	≈ 1

The table shows locus name, observed (H_*obs*_) and expected (H_*exp*_) heterozygosity, number of alleles (NA), Hardy–Weinberg Equilibrium probability (HWE), probability of identity, for each locus and for all loci, calculated with GENALEX and exclusion probabilities, associated with each locus and for all loci, calculated with GERUD.

All 20 artificial nests were occupied by territorial males. However, the genotype analysis of the fin-clips collected at the beginning and at the end of the experiment revealed 4 nest-takeover events. A different territorial male, indeed, was defending the nest at the end of the experiment, and so these nests have been excluded from the analysis. Paternity was assessed on 16 nests for a total of 1417 embryos (88.56 ± 7.30 embryos per nest). The average paternity success of territorial males was 72.37% ± 14.32, ranging from 41 to 91%, with an average number of estimated sneakers of 3.00 ± 0.73, ranging from 2 to 4, and of estimated mothers of 6.25 ± 1.53, ranging from 3 to 8.

#### Paternity success distribution

**Figure 3:**
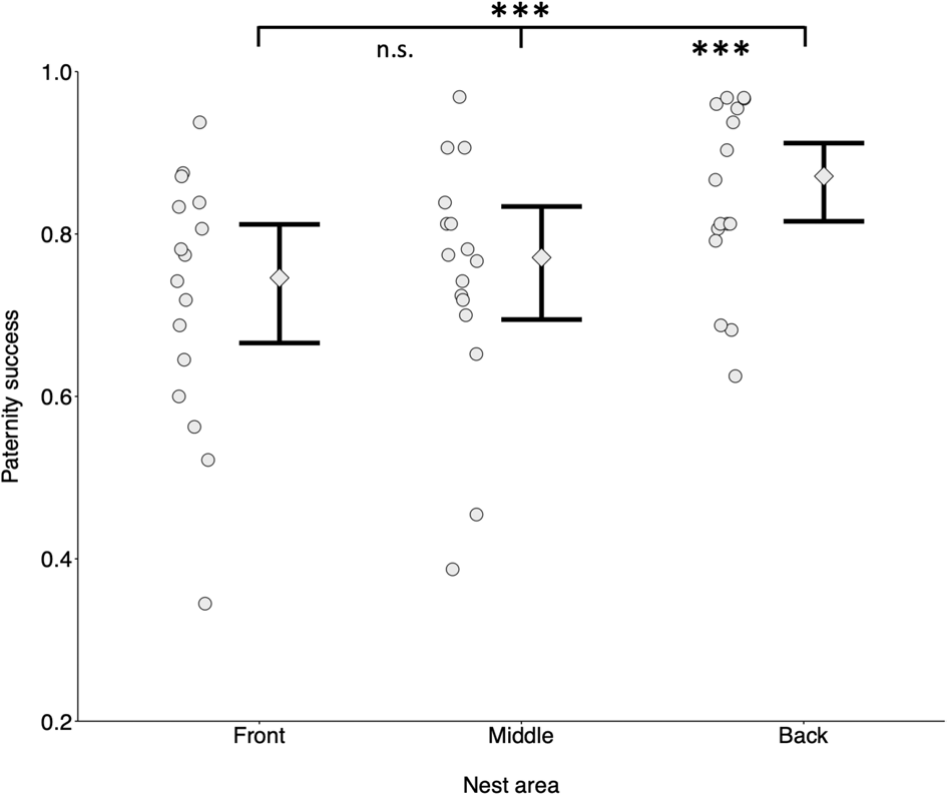
Proportion of the territorial males’ paternity success as a function of nest area (‘front’, ‘middle’, ‘back’). Territorial males achieve higher paternity rates in the back of the nest compared to the front and the middle. Data points: average males’ success for each nest (n = 16). Error bars: model-predicted group means (diamonds) with 95% credible intervals (vertical bold black lines) computed for each nest area over 10,000 simulations of the model and averaged across number of sneakers, number of females and sites.

Paternity success of territorial males decreased with increasing number of sneaker males and differed across nest areas (GLMM: number of sneakers p = 0.019, nest area p < 0.001, N = 16) (Fig. 3, Table 4). In particular, regardless the number of sneakers parasitizing the matings, the proportion of eggs fertilized by territorial males was higher in the back area (0.85 ± 0.11) of the nest compared to the front (0.72 ± 0.15) and the middle area (0.75 ± 0.15) (pairwise contrasts: front-middle p = 0.64; front-back p < 0.001; middle-back p < 0.001) (Fig. 3, Table 5).

**Table 4:** Statistical analysis: paternity success distribution.

Predictors	Predicted mean	Lower 95% CI	Upper 95% CI	P
**B. Paternity success of territorial males as a function of number of sneakers and nest area** **N = 16 nests, R**^**2**^_**cond**_** = 0.15**				
Intercept (front and site 1)	1.313	0.890	1.736	< 0.001
Nest area (middle and site 1)	0.138	− 0.161	0.437	0.366
Nest area (back and site 1)	0.840	0.501	1.178	< 0.001
Number of sneakers (standardised)	− 0.409	− 0.751	− 0.067	0.019
Number of females (standardised)	− 0.088	− 0.428	0.251	0.610
Breeding site (2)	− 0.447	− 0.957	0.064	0.087

Generalized linear mixed model with number of eggs fertilized by territorial and sneaker males as response variable. Predicted means and their credible intervals (CI) are based on a binomial distribution with logit-link in (see Materials and methods). For factorial predictors, estimates are computed using the indicated intercept level as reference. This choice is arbitrary and does not affect overall conclusions.

**Table 5:** Post-hoc tests of the analyses presented in Fig. 3 and Table 4.

Contrast	Odds ratio	Lower 95% CI	Upper 95% CI	P
**Pairwise differences in paternity success of territorial males between nests’ areas**				
Front versus middle	0.871	0.609	1.245	0.637
Front versus back	0.432	0.288	0.647	< 0.001
Middle versus back	0.496	0.329	0.747	< 0.001

Pairwise differences of the paternity success of territorial males between the three areas of the nests. Note that the contrasts are expressed as odds ratios, and averaged across the levels of site. Such ratios and their 95% confidence intervals (CI) are back transformed from the log odds ratio scale. If the confidence interval of the odd ratio excludes 1, the difference is significant. P values are corrected for multiple testing using the Tukey method.

## Discussion

Our results show that the intensity and the effectiveness of territorial males’ mate guarding and of the sneakers’ attempts differ between the front and the back entrances of the nest. The behavioural mating effort of both male types shows a similar trend: sneaking pressure and nest guarding are higher at the larger front access compared to the smaller one at the back. However, at the front entrance sneakers are more successful in overcoming territorial males defence and, as a result, the territorial male paternity success is lower in the area closer to this access. The opposite occurred at the back entrance, with territorial males being more successful in keeping sneakers away, gaining a higher fertilization success. In this species, sneakers are smaller than dominant males and mimic female coloration. Taking advantage of these characteristics, they gain proximity and quickly streak within territorial nests. Overall, the nest entrances appear to be differently vulnerable to sneaker intrusions, driving a spatially asymmetric distribution of the paternity share.

Until now, the influence of nest accessibility on territorial male paternity success has received little attention, though few studies have investigated the role of nest opening width without always finding a clean and clear relationship. By considering nest architecture we suggest that a great deal of interesting variation can be uncovered and explained. The size of nest accesses influences sneaking success in the cichlids *Lamprologus lemairii* and *Lamprologus callipterus*^22,23^, as narrow openings limit the intrusion of sneaker males. In particular, *Lamprologus callipterus* breed in empty gastropod shells where both large territorial males and middle size sneakers are forced to ejaculate far from the female that, being smaller, can enter and spawn in the shell. However, on rare occasions, small dwarf parasitic males may succeed in wriggling in shells, thus releasing sperm from the most favourable position^22,35^. By contrast, the width of nest entrances does not affect sneaking success in the sand goby, *Pomatoschistus minutus*, despite guardian males building smaller entrances as the number of sneakers increases^32,59^. Regardless the role played by the size of the nest accesses, the nests of the three mentioned species have a single entrance, which likely favours the efficacy of territorial males’ nest guarding. Overall, only a portion of their broods appears to be cuckolded, with guardian males siring on average 80% of the offspring in *Lamprologus lemairii*^23^, around 99% in the *Lamprologus callipterus*^22^, and 90% in the sand goby, *Pomatoschistus minutus*^60^. By contrast, in the black goby where nests present two openings we found that all territorial males experience a substantial degree of cuckoldry. Moreover, their paternity success is on average 72.37% (± 14.32%), which is low compared to other species with guard-sneaker tactics and a similar intensity of competition, i.e. two to four males participating to each spawning event^19–26^. In other species, where guardian males suffer high degree of cuckoldry and/or low paternity success the structure of the nest seems to favour opportunistic males’ intrusions. In the Mediterranean damselfish, *Chromis chromis*, spawning in exposed shallow pits, nests are always parasitized by two to seven sneakers and dominant male sire on average less than 50% of offspring^25^. In the grass goby, *Zosterisessor ophiocephalus*, nests are muddy chambers with several entrances and the offspring is always fathered by multiple males with the fertilization success of dominant males being on average 76.5%^21^. In the lingcod, *Ophiodon elongatus*, guardian males suffer cuckoldry in more than half of the nests, having an average paternity success of 78%, and the location of nest areas potentially vulnerable to sneakers’ intrusions can be inferred by the paternity distribution^27^.

Overall, nest structure may shape not only pre-mating strategies of territorial males to avoid rivals’ intrusions (i.e. nest defence), but also post-mating behaviours. In particular, because of the spatial asymmetry of paternity success, nest shape may work as an indirect cue of the uncertainty of paternity among different nest areas, thus influencing territorial males’ parental behaviour. In several fish species with ARTs, the uncertainty of paternity affects the parental effort of guardian males, tuning egg care or cannibalism in relation to sneaking pressure^15–18^. Territorial males may rely on direct (i.e. produced by the offspring) or indirect (i.e. derived by the environmental and social context) cues to assess their relatedness to the offspring and adjust their parental efforts^14,15,61–63^. In the black goby, the predictable spatial distribution of the paternity success might drive a selective cannibalism towards the embryos located in the front area of the nest, where their siring success is lower, and/or increasing care on the embryos in the back of the nests, where their paternity is higher. Preliminary experimental tests evaluating the parental behaviour of black goby territorial males exposed to the presence of two sneakers, showed that territorial males cannibalised eggs at a similar rate in all nest areas and parental cares were adjusted according to the number of embryos present^64^. However, these observations do not exclude the possibility that black goby territorial males may in fact use the paternity gradient to fine tune their post-mating behaviour. The reliability of this gradient, as an indirect cue of paternity loss, might depend on the intensity of sneaking pressure experienced by the territorial males while mating^61^. The information conveyed by the visual detection of two sneakers, not interfering with spawning, may be under the threshold to trigger the differential cannibalism among nest areas. Similarly, in the plainfin midshipman fish, *Porichthys notatus*^19^, the presence of a sneaker does not affect neither the intensity of parental care over the offspring nor the nest choice of territorial males^65^. By contrast, in a small Sulawesi fish, *Telmatherina sarasinorum*, where only the 12% of spawning events are cuckolded, the occurrence of one sneaker increases threefold the egg cannibalism^16^. In such a scenario, the deciphering of parental males’ decision making in response to change in mating contexts would benefit from the identification and the relationships of cues that are reliable indicator of paternity^61^.

Interestingly, in the black goby, embryo distribution significantly varies along the nest, with females laying more eggs in the middle and back areas than at the front one^64^. Females may prefer to lay eggs deeper in the nest to reduce the risk of predation, as egg mortality rate is generally higher close to the nest entrance^66–68^. However, the preference for specific areas of nests might also underlie a form of cryptic female choice, i.e. female ability to bias the fertilization success of males they mate with^69^. For instance, in the cooperative cichlid fish, *Julidochromis transcriptus*, polyandrous females carefully choose deposition sites that allow both large dominant males and small subordinate ones to fertilize their eggs, overall gaining parental care by all their mating partners^70^. In the black goby, females actively choose nesting males that guarantee cares to their eggs, but, being external fertilizers, they cannot avoid sneaker presence^38^. Thus, females might lay the eggs as far as possible from the front and most vulnerable area to favour the paternity of the dominant male.

Our results showed that spawning site structure influences the outcome of sneakers-dominant males’ competition for fertilization. Nest architecture, indeed, influences sneaking opportunities and the distribution of the territorial male paternity success. A predictable paternity distribution might also result in an indirect cue for guarding males to fine-tune their behavioural responses to optimize the investment towards their own offspring, as well as for females to lay eggs in the nest areas that better guarantee their mate choice.

## Supplementary Information

Supplementary Information.

## Data Availability

All data are available in the Dryad repository (https://doi.org/10.6084/m9.figshare.13356218)^57^.
