# Supplementary material for: Spatial asymmetry of the paternity success in nests of a fish with alternative reproductive tactics
Source: Sci Rep. 2021 Feb 4;11:3091. doi: 10.1038/s41598-021-82508-6 (PMC7862370; doi:10.1038/s41598-021-82508-6)
Supplement: Supplementary file 1 — Supplementary Information. [file 41598_2021_82508_MOESM1_ESM.docx]

*Spatial asymmetry of the paternity success in nests of a fish with alternative reproductive tactics*

*Poli, F. ^1*^, Marino, I.A.M. ^1, 2^, Santon, M.^3^, Bozzetta, E. ^1^, Pellizzato, G. ^1^, Zane, L. ^1, 2^, Rasotto, M. B. ^1^*

Supplementary Data

The size of artificial nests (14 cm x 14 cm x 3.5 cm) was estimated from measurements of natural nests obtained in the field (see the table below; N = 35). Moreover, the distance at which they were placed form each other was based on the average distance among natural nests (N = 35; average = 15.34 cm ± 3.39; min = 10 cm; max = 25 cm). Finally, the small access at the back that was dug by territorial males was in the size range of natural nests (N = 20; average = 3.08 cm ± 0.68; min = 2.00 cm; max = 4.50 cm).

| *Natural nests (N=35)* | | | | |
| --- | --- | --- | --- | --- |
|  | **mean** | **SD** | **min** | **max** |
| length | 14.30 | 2.52 | 10.00 | 20.00 |
| width | 14.14 | 1.72 | 11.00 | 18.00 |
| height of the back access (dug by males) | 3.16 | 1.72 | 2.00 | 5.50 |
| *Artificial nests (N=20)* | | | | |
|  | **mean** | **SD** | **min** | **max** |
| length | 14 | 0 | 14 | 14 |
| width | 14 | 0 | 14 | 14 |
| height of the back access (dug by males) | 3.08 | 0.68 | 2.00 | 4.50 |
